# Opposite-sex attraction in male mice requires testosterone-dependent regulation of adult olfactory bulb neurogenesis

**DOI:** 10.1038/srep36063

**Published:** 2016-10-26

**Authors:** Roberta Schellino, Sara Trova, Irene Cimino, Alice Farinetti, Bart C. Jongbloets, R. Jeroen Pasterkamp, Giancarlo Panzica, Paolo Giacobini, Silvia De Marchis, Paolo Peretto

**Affiliations:** 1 Department of Life Sciences and Systems Biology, University of Turin, Turin I-10123, Italy; 2 NICO–Neuroscience Institute Cavalieri Ottolenghi, University of Turin, Turin I-10125, Italy; 3 Inserm, UMR-S 1172, Laboratory of Development and Plasticity of the Neuroendocrine Brain, F-59000 Lille, France; 4 Department of Neuroscience, University of Turin, Turin I-10125, Italy; 5 Department of Translational Neuroscience, Brain Center Rudolf Magnus, University Medical Center Utrecht, 3584 CG11 Utrecht, The Netherlands; 6 Neuroscience Institute of Turin (NIT), University of Turin, Turin I-10125, Italy; 7 Univ. Lille, UMR-S 1172-JPArc-Centre de Recherche Jean-Pierre AUBERT Neurosciences et Cancer, F-59000 Lille, France

## Abstract

Opposite-sex attraction in most mammals depends on the fine-tuned integration of pheromonal stimuli with gonadal hormones in the brain circuits underlying sexual behaviour. Neural activity in these circuits is regulated by sensory processing in the accessory olfactory bulb (AOB), the first central station of the vomeronasal system. Recent evidence indicates adult neurogenesis in the AOB is involved in sex behaviour; however, the mechanisms underlying this function are unknown. By using Semaphorin 7A knockout (Sema7A ko) mice, which show a reduced number of gonadotropin-releasing-hormone neurons, small testicles and subfertility, and wild-type males castrated during adulthood, we demonstrate that the level of circulating testosterone regulates the sex-specific control of AOB neurogenesis and the vomeronasal system activation, which influences opposite-sex cue preference/attraction in mice. Overall, these data highlight adult neurogenesis as a hub for the integration of pheromonal and hormonal cues that control sex-specific responses in brain circuits.

The regulation of reproductive physiology in rodents involves the integration of salient chemosensory stimuli (i.e., pheromones) with endocrine signals^1^,^2^. In particular, olfactory cues appear crucial for the expression of ‘appetitive’ or pre-copulatory sexual behaviours^3^,^4^, which include the investigation of and approach towards the opposite sex. Appropriate reproductive responses to pheromones are triggered by the action of gonadal hormones on brain circuits, whose sex-specific organization is established early during a developmental critical period^3^,^5^. These neural circuits include several nuclei of the vomeronasal system (VNS) and hypothalamus, and the integration of pheromonal and endocrine signals in these circuits is required to regulate the activity of the hypothalamic-pituitary-gonadal (HPG) axis and thus reproduction^6^. Pheromones and sex hormones are also key regulators of adult neurogenesis, the process of the continuous generation of new neurons that occurs in restricted regions of the adult brain, namely the olfactory bulb (OB) and the dentate gyrus of the hippocampus^7^,^8^. In the accessory olfactory bulb (AOB; i.e., the first relay station in the VNS) of female mice, adult neurogenesis is positively regulated by male pheromonal stimuli and is essential for one of the best-known examples of neuroendocrine responses elicited by pheromones: the exteroceptive block of pregnancy or Bruce effect^9^,^10^,^11^. In addition, pheromonal perception in both sexes, as well as pregnancy and lactation in females, drives the secretion of adenohypophyseal hormones (e.g., prolactin and luteinizing hormone) and sex steroids (e.g., oestradiol and testosterone -TST), which in turn influence adult neurogenesis^12^,^13^,^14^,^15^. Thus, increasing evidence points to a role for adult neurogenesis in the control of reproduction through the integration of pheromonal and sex hormonal cues^8^,^16^,^17^, yet the mechanisms underlying this integration remain largely elusive. To address this issue, here, we investigated adult AOB neurogenesis and activation of the VNS in the context of pre-copulatory sexual behaviour in Sema7A ko mice^18^. These animals are characterized by defective migration of gonadotropin releasing hormone (GnRH) neurons during development, which results in a significant reduction of GnRH neurons in the adult hypothalamus as well as reduced testis size and subfertility^19^. GnRH neurons are the master regulators of reproductive functions in all vertebrates and orchestrate the HPG axis by integrating multiple sensory signals, including olfactory cues^20^,^21^,^22^. Accordingly, defective GnRH function is related to abnormal olfactory and reproductive responses^23^. Here, we show that in the Sema7A ko males, as well as in wild-type males castrated during adulthood, the level of circulating TST in adult animals plays a critical role in modulating pheromone-induced adult neurogenesis in the AOB and, in turn, neuronal activity in downstream VNS nuclei that control sex-specific reproductive responses. These results indicate that the expression of suitable sexually dimorphic responses to opposite-sex pheromones requires fine-tuned cooperation between gonadal hormones and adult neurogenesis.

## Results

### Sema7A ko male mice show reduced circulating testosterone levels and altered opposite-sex odour preference

Sema7A ko mice show a reduced number of GnRH neurons, reduced testis size and subfertility^19^. Here, to assess the impact of this phenotype on olfactory-dependent reproductive behaviour, we first evaluated the concentration of blood TST and the expression of gonadal hormone receptors in the OB of adult ko males. Indeed, appropriate levels of circulating TST and gonadal hormone receptor expression are required to sustain the typical male preference for female odours^24^,^25^,^26^,^27^.

**Figure 1:**
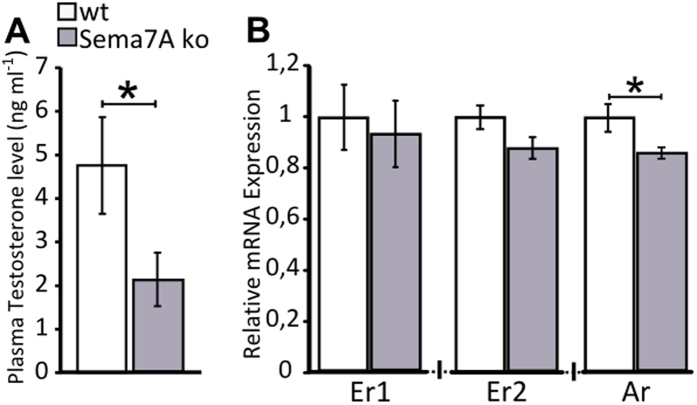
Reduced testosterone levels and androgen receptor expression in Sema7A ko male mice. (**A**) Plasma testosterone (TST) levels in wt and Sema7A ko male mice (n = 8 wt, 8 ko; unpaired Student’s t-test, P = 0.024) indicate a reduction in TST levels in ko mice. (**B**) Real-time PCR analysis of oestrogen receptor (Er 1 and 2) and androgen receptor (Ar) expression in the olfactory bulb, normalized to values in wt mice (=1), indicates decreased Ar receptor expression in Sema7A ko mice (n = 8 wt, 8 ko; Wilcoxon-Mann-Whitney test, P = 0.015) and no differences in Er expression (Wilcoxon-Mann-Whitney test, P > 0.05). The values shown are the mean ± s.e.m.

We found a strong decrease in circulating TST levels in Sema7A ko compared to wild-type (wt) mice (Fig. 1A). TST acts either directly, via binding to androgen receptor (Ar), or through its aromatization, by activating Er1 and Er2 oestrogen receptors^28^,^29^. Using real-time PCR, we found a significant reduction of Ar expression in the OB of the Sema7A ko mice (Fig. 1B), whereas the expression of the oestrogen receptor-encoding genes Er1 and Er2 did not significantly differ between the two genotypes (t-test P > 0.05; Fig. 1B).

**Figure 2:**
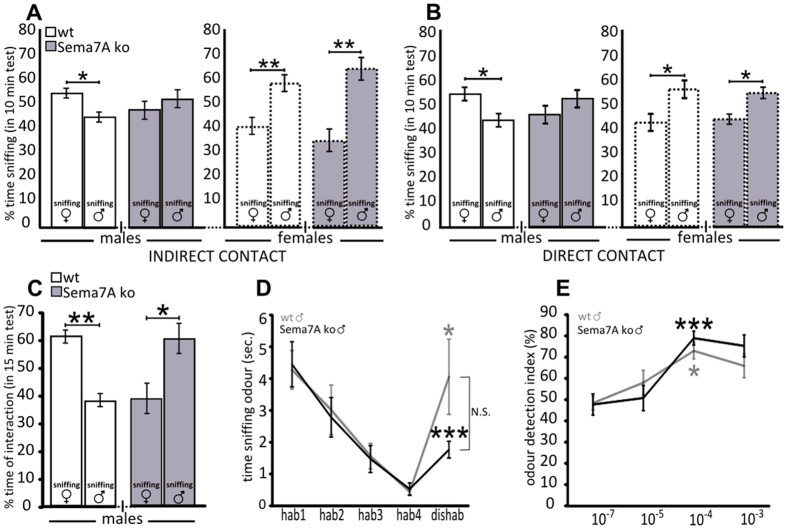
Altered opposite-sex responses in Sema7A ko males. (**A**) During indirect contact with pheromones (volatile compounds), wt male mice spend more time sniffing female odours (n = 8; Wilcoxon-Mann-Whitney test, P = 0.015), while Sema7A ko males show no preference (n = 8; Wilcoxon-Mann-Whitney test, P > 0.05). Both wt and Sema7A ko females spend more time in contact with the odour of the opposite sex (n = 8 wt, 8 ko; Wilcoxon-Mann-Whitney test, P = 0.001). (**B**) The same behaviours are observed during direct contact with the pheromonal source: the wt males and females and Sema7A ko females spend more time in contact with the opposite-sex odours (n = 8; Wilcoxon-Mann-Whitney test, P = 0.020 for wt males; P = 0.015 for wt females; P = 0.011 for ko females), while the Sema7A ko males do not show any preference (n = 8; Wilcoxon-Mann-Whitney test, P > 0.05). (**C**) In the individual preference test, the wt males prefer the opposite sex (female) (n = 8; Wilcoxon-Mann-Whitney test, P = 0.0003), whereas the Sema7A ko males spend more time in contact with the same sex (male) (n = 8; Wilcoxon-Mann-Whitney test, P = 0.021). (**D**) The habituation/dishabituation paradigm indicates that Sema7A ko males (black line) are able to discriminate habituation odours from dishabituation odours (n = 8; paired Student’s t-test, P = 0.0001), as observed in wt mice (grey line; n = 8; paired Student’s t-test, P = 0.014). No statistical significant difference (N.S.) is evident between the dishabituation time-points of Sema7A ko and wt mice (unpaired Student’s t-test, P = 0.08). (**E**) Odour detection threshold test shows that both Sema7A ko (black line) and wt mice (grey line) can detect odour (octanal) starting from 10^−4^ concentration (Sema7A ko, n = 7; paired Student’s t-test, P = 0.0004; wt, n = 8; paired Student’s t-test, P = 0.014). Normalized values are expressed as the mean ratio between the time spent investigating the odour and the total sniffing time (odour plus oil). The values shown are the mean ± s.e.m.

Next, the opposite-sex preference of Sema7A ko mice was evaluated by comparing the responses to urinary scents/pheromones from sexually active males versus sexually receptive females. This was done by exposing the mice to the airborne volatiles of familiar urine only, and after direct nasal-urine contact. Indeed, it is known that sex recognition through the main olfactory system (airborne volatiles) in female mice requires earlier activation of both vomeronasal and olfactory epithelia through urine direct contact (contact with familiar urine)^30^, implying a functional crosstalk between the two olfactory systems. As expected, wt males spent more time investigating urine from females (Fig. 2A,B). This was true both after exposure to the airborne volatiles of the familiar urine only (indirect contact, Fig. 2A) and after direct nasal-urine contact (Fig. 2B). By contrast, the Sema7A ko males never showed any preference for male or female scents after either indirect or direct contact with familiar urine (Fig. 2A,B), although the behaviour of the Sema7A ko females did not differ from that of wt females (Fig. 2A,B). These results point to a sexually dimorphic role of Sema7A for opposite-sex preference. Indeed, the lack of Sema7A affected the preference for opposite-sex odour in males but not in females. The response of males was further investigated using a three-chamber social interaction assay^31^ in which the subject can interact directly with two stimulus animals. Again, wt males spent more time interacting (sniffing and contacting) with receptive females, whereas the Sema7A ko males showed significant preference to interact with the male (Fig. 2C), further supporting an altered sexual preference in ko males.

Because the main olfactory system has been shown to participate in sex discrimination^32^, we conducted habituation-dishabituation olfactory tests and odour detection threshold analyses to measure main olfactory epithelium-mediated olfactory discrimination^33^,^34^ using non-social odour stimuli (i.e., acetophenone and octanal; Fig. 2D,E). These experiments did not show any significant impairment in the odour discrimination and olfactory sensory perception of Sema7A ko males (Fig. 2D,E), suggesting that the alteration in sex-preference in Sema7A ko males is unlikely to depend on the main olfactory system. The lack of defects in olfactory sensory perception in these animals is further supported by the lack of difference between ko versus wt males in the total time of sniffing of urinary scents (Suppl. Fig. 1A). Similarly, no differences were found when considering the total time of individual interaction in the two groups of males (Suppl. Fig. 1D).

Overall, these data suggest that altered opposite sex-preference in Sema7A ko males is not attributable to a general reduction of either olfactory sensory perception or motivation to explore social stimuli, but rather can imply the involvement of the accessory olfactory system.

### Feminized neurogenic response to male pheromones in the AOB of Sema7A ko males

**Figure 3:**
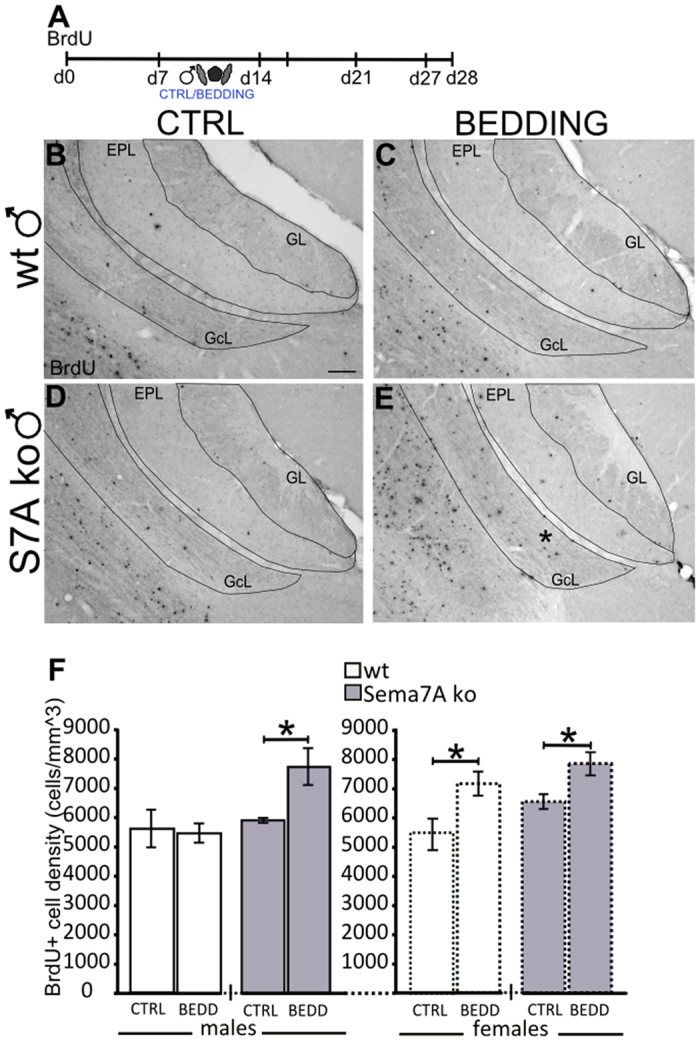
Sema7A ko males display feminized-like neurogenesis in response to male pheromones. (**A**) Experimental protocol: newborn cells were quantified in the GcL of the AOB 28 days after BrdU injection. From day 7 to day 14, the mice were familiarized with male-soiled bedding, while the control groups received clean bedding. (**B–E**) Representative images of AOB sections showing BrdU-positive newborn neurons in wt (**B,C**) and Sema7A ko (**D,E**) males after bedding familiarization. (**F**) Quantification of BrdU-positive cell density in the AOB GcL indicates an increase in newborn neurons in the Sema7A ko males after male-soiled bedding (BEDD) exposure compared to the clean bedding (CTRL) condition (n = 4; unpaired Student’s t-test, P = 0.030); no differences are observed in wt males (n = 4 CTRL, n = 6 BEDD; unpaired Student’s t-test, P > 0.05). Both wt and Sema7A ko females show increased BrdU-positive cell density in the BEDD condition (n = 4 wt, 4 ko) compared to the CTRL condition (n = 4 wt, 5 ko; unpaired Student’s t-test, P = 0.043, P = 0.024, respectively). The values shown are the mean ± s.e.m. Abbreviations: AOB: accessory olfactory bulb; GcL: granule cell layer; GL: glomerular layer; EPL: external plexiform layer; S7A: Semaphorin 7A; BrdU: bromodeoxyuridine. Scale bar: 100 μm.

Work by our and other laboratories has demonstrated that exposure to male chemosensory stimuli increases adult neurogenesis in female rodents, optimizing intersexual interaction^11^,^13^,^35^,^36^. In wt female mice, one-week exposure to stud-male pheromones (familiarization) promotes the survival/integration of newborn granule cells in the AOB^10^,^11^. Notably, this effect was not found in males^11^. Based on the altered (feminized) preference to opposite-sex cues observed in the Sema7A ko males, we evaluated adult neurogenesis in the OB of these animals. The number of newborn cells was quantified 28 days after 5-bromo-2′-deoxyuridine (BrdU) injection in mice exposed for 1-week to either clean bedding or stud-male urine-soiled bedding, following a previously established experimental paradigm that allows to evaluate newborn cell survival in the AOB^11^ (Fig. 3A). The clean bedding condition revealed no statistically significant difference in the density of BrdU-positive cells in the AOB of ko versus wt males (Fig. 3B,D,F). Similarly, no difference was found in the main olfactory bulb (MOB; Suppl. Fig. 2A). Moreover, unchanged rate of cell proliferation was observed in the SVZ-RMS of Sema7A ko, evaluated at two hours after a single BrdU pulse (Suppl. Fig. 2B). These data indicate that in the basal condition (absence of stud-male pheromones), a normal rate of newborn cell proliferation/survival occurs in the olfactory system of Sema7A ko males. By contrast, the ko males exposed to stud-male pheromones showed increased survival of BrdU + granule cells in the AOB (Fig. 3D–F), without any change in the AOB granule cell layer (GcL) volume (wt: 0.0421 ± 0.003 mm^3^; Sema7A ko: 0.0451 ± 0.003 mm^3^; unpaired Student’s t-test, P = 0.5). Notably, this increase in newborn cell density was comparable to that found in wt and ko females exposed to male pheromones (Fig. 3F). In contrast, these cues did not enhance AOB neurogenesis in wt males (Fig. 3B,C,F), or in the MOB of both wt and ko males (Suppl. Fig. 2C). Overall, these findings show that the increase of newborn neurons in the Sema7A ko males is associated with an effect of male soiled bedding in cell survival specifically in the AOB, indicating Sema7A ko males exhibit a feminized AOB neurogenesis phenotype as well as a feminized sexual preference phenotype.

### The male cue-elicited c-fos expression pattern along the vomeronasal pathway is feminized in Sema7A ko males

In female mice, exposure to the pheromones of familiar males increases the percentage of newborn AOB granule cells that express the immediate early gene c-fos^11^, which is a marker of neuronal activation^37^,^38^. In turn, the same stimuli elicit a sex-specific c-fos expression pattern in the downstream nuclei of the VNS^11^,^39^,^40^, wherein the integration of pheromonal and endocrine signals modulates opposite-sex attraction/preference^6^,^41^. Therefore, considering the feminized responses to male pheromones observed in the Sema7A ko males, both at the behavioural and cellular level, we investigated c-fos expression in the VNS of these mice.

**Figure 4:**
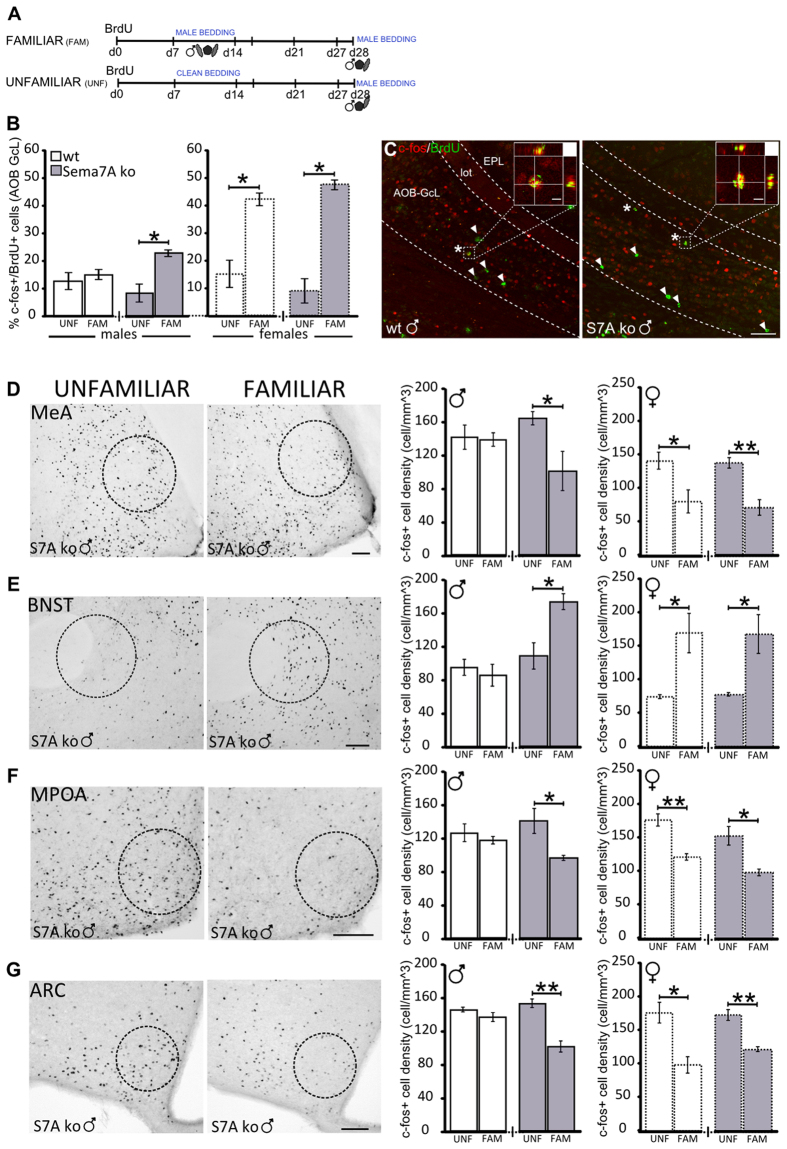
The vomeronasal pathway of Sema7A ko males shows a feminized c-fos response to pheromones. (**A**) Experimental protocol: the familiar groups were stimulated with male-soiled bedding during the 2nd week after BrdU administration, from day 7 to day 14, whereas the unfamiliar groups received clean bedding. Both groups were then stimulated with male bedding 90 min before animal perfusion. (**B**) Quantification of c-fos/BrdU double-labelled cells in the AOB GcL reveals an increase in the familiar Sema7A ko group compared to the unfamiliar Sema7A ko group (n = 4; Wilcoxon-Mann-Withney test, P = 0.028). No differences are observed in wt males (n = 4, Wilcoxon-Mann-Withney test, P > 0.05). An increase in c-fos/BrdU+ cells is also observed in the familiar versus unfamiliar group of wt females (n = 4; Wilcoxon-Mann-Withney test, P = 0.029) and Sema7A ko females (n = 4; Wilcoxon-Mann-Withney test, P = 0.028). (**C**) BrdU (green) and c-fos (red) immunofluorescence in the AOB-GcL of wt and Sema7A ko males. Asterisks indicate representative c-fos/BrdU double-stained cells. Arrowheads indicate c-fos-negative/BrdU-positive cells. Scale bars: main panels 50 μm; magnified panels 5 μm. (**D–G**) Representative images of Sema7A ko males and histograms showing changes in c-fos expression throughout the VNS in Sema7A ko and wt mice after bedding familiarization. The quantification of c-fos+ cell density shows no differences between the unfamiliar and familiar groups of wt males (unpaired Student’s t-test, P > 0.05). In Sema7A ko males and in wt and Sema7A ko females, familiarization decreases c-fos cell density in the medial amygdala (MeA, **D**), medial preoptic area (MPOA, **F**) and arcuate nucleus (ARC, **G**) and increases c-fos expression in the bed nucleus of the stria terminalis (BNST, **E**). (Sema7A ko males n = 3; MeA P = 0.034; BNST P = 0.024; MPOA P = 0.044; ARC P = 0.003; wt females n = 3; MeA P = 0.047; BNST P = 0.033; MPOA P = 0.006; ARC P = 0.016; Sema7A ko females n = 3; MeA P = 0.009; BNST P = 0.037; MPOA P = 0.020; ARC P = 0.004). The values shown are the mean ± s.e.m. Scale bar: 100 μm.

In Sema7A ko males, focal exposure (30-min bedding/urine presentation 90 min before sacrifice, Fig. 4A) to previously experienced (familiar) male cues induced a net increase in the percentage of c-fos/BrdU-positive newborn AOB neurons in comparison to focal exposure with unfamiliar cues (Fig. 4B,C). By contrast, wt males did not show any difference in c-fos expression in response to familiar versus unfamiliar male cues (Fig. 4B). As expected, in wt females, we found an increase in c-fos expression in response to familiar male cues (Fig. 4B), and a similar pattern was also found in the Sema7A ko females (Fig. 4B).

In the VNS nuclei downstream of the AOB, the Sema7A ko males showed an increase in c-fos-positive cells in the bed nucleus of the stria terminalis (BNST, Fig. 4E), while the number of c-fos positive cells was reduced in the medial amygdala (MeA), medial preoptic area (MPOA), and arcuate nucleus (ARC) (Fig. 4D,F,G). In wt males, there was no difference in c-fos expression in response to familiar versus unfamiliar male cues in these nuclei (Fig. 4D–G), whereas both wt and Sema7A ko females showed the same pattern observed in the Sema7A ko males (Fig. 4D–G). Thus, the lack of Sema7A impacts male sexual behaviour, possibly through abnormal (feminized) neuronal activation along the VNS pathway.

### Testosterone administration reverses the changes in sex-preference, neurogenic and VNS c-fos responses in Sema7A ko mice

**Figure 5:**
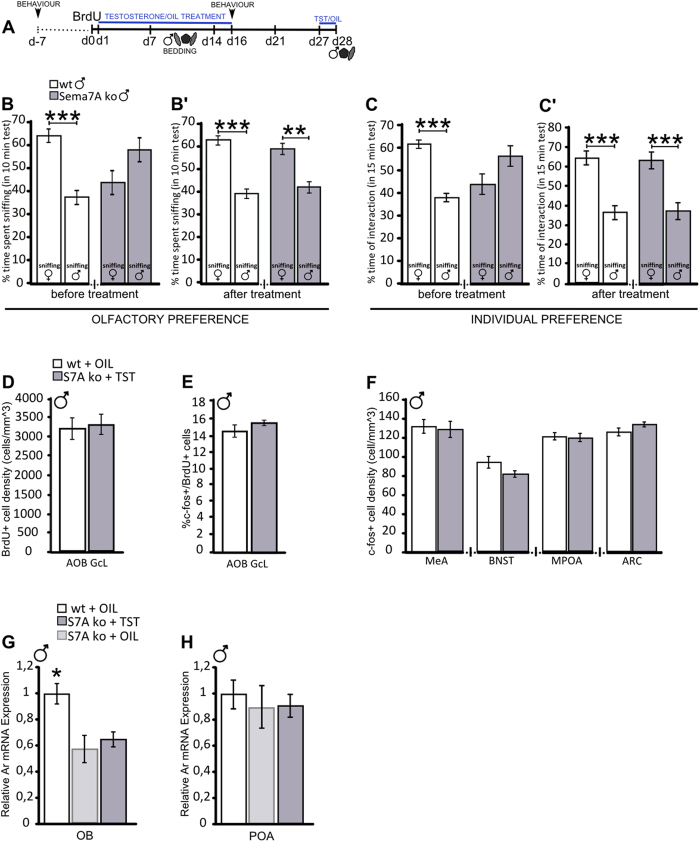
Administration of testosterone in Sema7A ko male mice restores opposite-sex preferences, neurogenic activity and the c-fos response to pheromones. (**A**) Experimental protocol: testosterone (TST; for Sema7A ko) or oil (for wt) was administered for 16 days starting one day after BrdU injection (d1–d16). Two additional pulses were given 2 days before animal perfusions. The mice were familiarized with male bedding between d7–d14 after BrdU injection, then received a focal stimulus with male bedding 90 min before perfusion. Behaviour was assessed one week before, and at the end of the TST/oil treatment (d16). (B-B’) Olfactory preference behaviour. Before treatment (**B**), wt mice prefer female odours (n = 7; Wilcoxon-Mann-Whitney test, P = 0.0006), while Sema7A ko males do not show any preference (n = 7; Wilcoxon-Mann-Whitney test, P > 0.05). After treatment (B’), both wt and Sema7A ko males show a preference for the opposite sex (Wilcoxon-Mann-Whitney test, wt P = 0.0006; ko P = 0.001). (C-C’) Individual preference behaviour. Before treatment (**C**), wt mice prefer female odours (n = 7; Wilcoxon-Mann-Whitney test, P = 0.0006), while Sema7A ko males do not show any preference (n = 7; Wilcoxon-Mann-Whitney test, P > 0.05). After treatment (C’), both wt and Sema7A ko males show a preference for females (Wilcoxon-Mann-Whitney test, wt P = 0.0006; ko P = 0.0006). (**D**) After treatment, no difference in BrdU+ cell density is observed between the wt and Sema7A ko mice (n = 6 wt, 6 ko; unpaired Student’s t-test, P > 0.05). (**E**) After treatment, no difference in cfos/BrdU+ cell percentage is observed between the wt and Sema7A ko males (n = 4 wt, 4 ko; Wilcoxon-Mann-Whitney test, P > 0.05). (**F**) After treatment, no differences in cfos+ cell density in the medial amygdala (MeA), bed nucleus of the stria terminalis (BNST), medial preoptic area (MPOA) and arcuate nucleus (ARC) were found between the wt and Sema7A ko males. (**G,H**) Real-time PCR analysis indicates that TST administration does not restore the level of androgen receptor (Ar) in the OB of ko males (n = 4wt, 4Sema7A, 5Sema7A + TST; Kruskall-Wallis Test; P = 0.023). In the preoptic area (POA) no differences are visible between wt and oil- or TST-treated ko males (n = 4wt, 3Sema7A, 4Sema7A + TST; Kruskall-Wallis Test; P > 0.05). The values are normalized to wt mice ( = 1). The values shown are the mean ± s.e.m.

We speculated that the sex-specific pheromonal-dependent response observed in the Sema7A ko males at both the behavioural and cellular levels could be directly related to the reduced circulating TST found in these animals. To test this hypothesis, one cohort of adult ko males received, in addition to BrdU injection and bedding familiarization, a TST replacement treatment^42^ that involved daily subcutaneous injections of TST proprionate (Fig. 5A). One week before the TST treatment, the Sema7A ko males showed a feminized sex-preference response to opposite-sex scents or individuals, as expected (Fig. 5B,C). Sixteen days of TST treatment in these animals was sufficient to restore the typical preference for opposite-sex stimuli (Fig. 5B’,C’). This occurred without changes in the total time of sniffing in olfactory and individual preference tests (Suppl. Fig. 1B,E), suggesting that the level of circulating TST mediates opposite-sex preference without affecting sensory perception or motivation to investigate social stimuli. In addition, TST administration did not modify the level of Ar expression neither in the OB, where a decrease was observed in the Sema7A ko (Figs 1B and 5G), nor in the hypothalamic preoptic area, which includes several nuclei involved in the control of sex behaviour^6^, and where no difference in Ar expression between wt and ko males were found (Fig. 5H).

Strikingly, following familiarization with male pheromones, the density of newborn cells in the AOB of the TST-treated Sema7A ko mice at 28 days after BrdU injection was similar to that in wt (oil-treated) males (Fig. 5D, Suppl. Fig. 3A). In line with these findings, we did not observe any difference in the pattern of c-fos expression in newborn AOB neurons or in downstream VNS nuclei between the TST-treated Sema7A ko males and wt males (Fig. 5E,F and Suppl. Fig. 3B–F). These data indicate that the feminized phenotype observed in the Sema7A ko males is fully reversible by TST treatment in adult life. In addition, they reveal a link between levels of circulating TST, the sex-dependent modulation of newborn cell survival in the AOB, and cellular responses in VNS nuclei involved in the control of sexual behaviour.

### Testosterone inhibits the neurogenic response to male pheromones in the AOB

**Figure 6:**
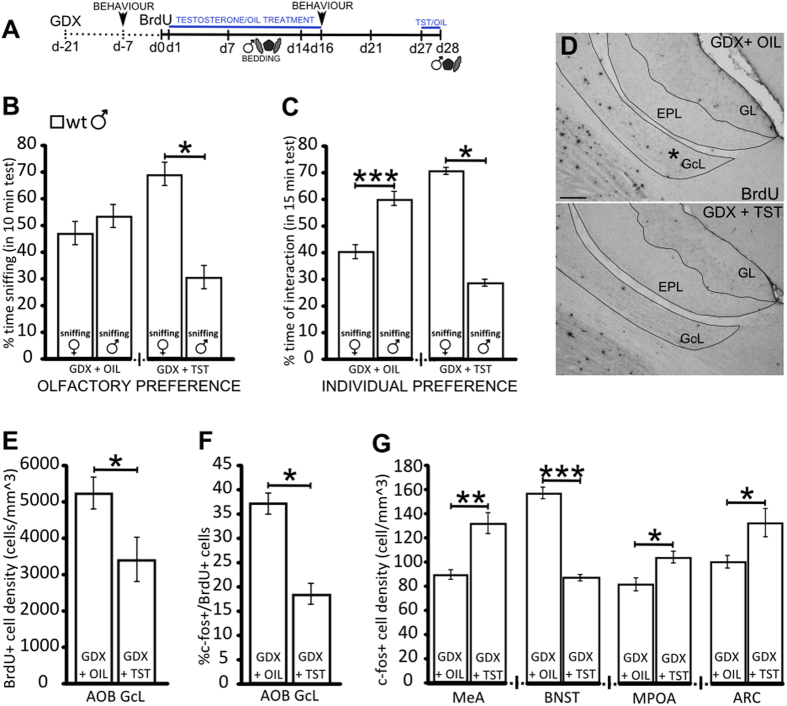
Gonadectomy and testosterone administration in wt mice mimic the behavioural and cellular responses that characterize the phenotype of the Sema7A ko males. (**A**) Experimental protocol: wt males were gonadectomized (GDX) 3 weeks before BrdU administration. Testosterone (TST)/oil treatment was administered for 16 days (d1–d16) starting one day after BrdU injection. Two additional pulses were given 2 days before animal perfusions. Male bedding was given the 2nd week after BrdU injection (d7–d14), and as a focal stimulus 90 min before perfusion. The behaviour of the GDX mice was assessed one week before TST/oil treatment and at the end of the treatment (d16). (**B**) Olfactory preference behaviour indicates no preference between female and male odours in the oil-treated GDX wt males (n = 10; Wilcoxon-Mann-Whitney test, P > 0.05), whereas the TST-treated GDX wt males show a preference for female odours (n = 4; Wilcoxon-Mann-Whitney test, P = 0.028). (**C**) Individual preference behaviour analysis shows oil-treated GDX males spend more time sniffing male than female odours (n = 10; Wilcoxon-Mann-Whitney test, P = 0.0002), while TST-treated GDX males prefer female than male odours (n = 4; Wilcoxon-Mann-Whitney test, P = 0.028). (**D**) AOB sections of oil- and TST-treated GDX wt mice showing the increase in BrdU+ cells in the AOB GcL of oil-treated animals. (**E**) Quantification of BrdU+ cell density in the AOB GcL shows increased newborn neuron density in oil-treated compared to TST-treated GDX wt mice (n = 6 oil treated, 4 TST treated; unpaired Student’s t-test, P = 0.036). (**F**) Quantification of c-fos/BrdU+ cells shows an increase in oil-treated compared to TST-treated GDX wt mice (n = 6 oil treated, 4 TST treated; Wilcoxon-Mann-Whitney test, P = 0.014). (**G**) Oil-treated GDX wt male mice show a lower density of c-fos+ cells in the MeA, MPOA and ARC and a higher density in the BNST compared to TST-treated GDX wt animals (n = 6 oil treated, 4 TST treated; unpaired Student’s t-test, MeA P = 0.002; BNST P = 0.0006; MPOA P = 0.019; ARC P = 0.044). The values shown are the mean ± s.e.m. Abbreviations: GcL: granule cell layer; GL: glomerular layer; EPL: external plexiform layer; MeA: medial amygdala; BNST: bed nucleus of the stria terminalis; MPOA: medial preoptic area; ARC: nucleus arcuatus;. Scale bar: 100 μm.

To further elucidate the role of TST in regulating sex-specific pheromonal-dependent responses in adult animals, we performed the same set of experiments in a cohort of wt males that were castrated during adulthood (and thus had normally developed sex-related brain circuits) and treated with TST or vehicle (sesame oil) alone (Fig. 6A). The oil-treated castrated males showed reduced or no interest in opposite-sex urine scents or individuals, whereas the TST-treated castrated males exhibited a clear preference for the opposite-sex cues, behaving as control males (Fig. 6B,C). Again, also in these groups no differences were found in the total time of sniffing in olfactory and individual preference (Suppl. Fig. 1C,F), further supporting these aspects are not related to the level of circulating TST during adulthood.

Notably, familiarization with male pheromones induced increased neurogenesis in the AOB of castrated oil-treated males in comparison to the TST-treated castrated males (Fig. 6D,E), indicating that in males, TST is necessary and sufficient to inhibit the AOB neurogenic response elicited by male pheromones. In line with these results, the c-fos expression pattern observed in the newborn AOB neurons and in the downstream nuclei of the VNS of the oil-treated castrated males under familiar male odour exposure was reminiscent of that observed in the Sema7A ko mice and significantly different from that in the TST-treated castrated males (Fig. 6F,G, and Suppl. Fig. 3). Overall, these data indicate that the level of circulating TST during adulthood is critical for the fine-tuned and sex-specific control of AOB neurogenesis and VNS activation, which influences opposite-sex cue preference/attraction.

## Discussion

In mammals, pheromones and gonadal hormones are known to work in concert to elicit sex-specific patterns of neuronal activity in the brain circuits that control sexual behaviour. In addition, studies conducted over the last decade in rodents have shown that correct pheromonal perception requires the integration and activity of newborn interneurons in the AOB, the first central station of the VNS pathway. Here, we demonstrate that in male mice, the level of circulating TST plays a critical role in regulating the process of adult neurogenesis in the AOB and, in turn, the pattern of neuronal activity in the downstream VNS nuclei that drives attraction towards females. Overall, these results strongly suggest that cooperation between the endocrine system and adult neurogenesis is needed for appropriate responses to social reproductive stimuli.

### Sema7A ko males show feminized behavioural and c-fos responses to opposite-sex stimuli

In vertebrates, GnRH neurons control reproduction by directing the activity of the HPG axis through the integration of a multitude of external and internal stimuli^20^. Accordingly, defects in the organization and/or function of the GnRH system cause heterogeneous reproductive disorders that are characterized by a reduction or failure of sexual competences^43^,^44^. In Sema7A ko males, a suboptimal number of GnRH neurons leads to impaired testicular growth^19^, which we found to be associated with lowered levels of circulating TST, reduced androgen receptor expression in the OB, and the absence of a preference/attraction towards opposite-sex smells and/or individuals that is not associated to altered olfactory perception and/or attraction to social cues. In mice, attraction to the opposite sex is mediated by pheromonal perception^45^ and is considered part of appetitive, or pre-copulatory, sexual behaviour^3^,^4^,^46^. The neural circuits responsible for sex behaviour involve several brain areas where the integration of olfactory input and endocrine stimuli occurs^6^,^41^. Importantly, in these circuits, pheromonal exposure triggers a sexually dimorphic pattern of excitation^25^,^39^,^40^,^47^,^48^,^49^,^50^. Here, we demonstrated that in Sema7A ko males, exposure to male cues elicited feminized c-fos responses in the AOB, MeA, BNST, MPOA, and ARC, which are target nuclei of the vomeronasal pathway involved in the control of sexual behaviour^39^,^40^,^51^. This pattern of c-fos expression is thus consistent with the altered attraction to opposite-sex stimuli found in the ko males. By contrast, in the Sema7A ko females, male pheromonal exposure elicited attraction and the expected c-fos expression pattern in sex-specific brain nuclei, indicating that in Sema7A ko mice, altered responses to opposite-sex stimuli occur only in males.

### Sexually dimorphic Testosterone-dependent regulation of AOB neurogenesis

One key point of our study is that in the Sema7A ko males, exposure to male pheromones increased the integration/survival of newborn interneurons in the AOB, a phenomenon that is normally restricted to females^10^,^11^ and that is upstream of the cascade of sexually dimorphic activation patterns that take place in the VNS nuclei^11^,^39^,^40^,^51^. Strikingly, in adult Sema7A ko males, chronic TST treatment was sufficient to block the effects elicited by male pheromonal exposure, repressing the increase in adult AOB neurogenesis and reversing the feminized phenotype, in terms of both the c-fos expression pattern along the VNS pathway and appetitive behaviour. Although we did not examine whether this effect is purely androgen dependent or mediated by the TST metabolites oestradiol and/or dihydrotestosterone, our data clearly indicate that the sexually dimorphic integration of newborn neurons in the AOB of adult mice involves a gonadal-dependent mechanism.

Altered sexual behaviour, including a shift of cellular and behavioural responses to opposite-sex pheromones, has been previously described in male mice with genetic alteration/elimination of androgen or oestrogen signalling^24^,^26^,^27^,^29^,^52^,^53^,^54^,^55^,^56^,^57^. Activation of these signalling pathways during the perinatal critical period is fundamental for brain masculinization and depends on a perinatal surge of TST from the testis and its conversion to oestrogens^3^,^5^, although activation of the Ar is also required^29^. Thus, it is conceivable that the low circulating TST level, as well as the reduction of Ar expression observed in the OB in the Sema7A ko mice could affect the perinatal organization of male sex-specific brain circuits and, in turn, attraction to females during adulthood. However, sexual motivation in male mice, which is related to pre-copulatory behaviour, also depends on the level of circulating TST during adulthood^58^,^59^,^60^. Accordingly, TST administration in adult Sema7A ko males was sufficient to restore the typical male pattern of cellular and behavioural responses along the VNS, although it was inefficient in restoring a normal level of Ar expression in the OB.

The castration of adult animals allows organizational effects to be distinguished from activational effects, which can be reversed by replacement of the active steroid^5^. Using this strategy in wt mice, we further demonstrated that an appropriate level of circulating TST during adulthood is both necessary and sufficient to modulate AOB neurogenesis and the activity of downstream pheromonal-sensitive brain circuits that control opposite-sex attraction/preference in male mice.

### Fine-tuned gonadal-dependent regulation of AOB neurogenesis controls opposite-sex attraction in mice

The role of the AOB as one of the primary integrative regions for sensory stimuli controlling reproduction has been well established by several studies^2^. AOB neurons encode the sexual and genetic status of conspecifics based on signals contained in body fluids, such as urine and saliva^30^,^61^, and their activity is required for several neuroendocrine responses to opposite-sex pheromones, such as the selective block of pregnancy or “Bruce effect”^9^. In addition, and of particular interest considering our results, in males, the activation of AOB circuits is important for the encoding of the motivational value of opposite-sex chemo-signals^60^,^62^. Key elements in the AOB sensory processing are the inhibitory interneurons, the granule and periglomerular cells, which gate excitatory inputs from the AOB that are sent to downstream nuclei of the VNS and hypothalamus. In both the MOB and AOB, these cells are continuously refreshed by the addition of ‘young and excitable’ elements, which become involved in sensory perception soon after their activity-dependent integration^11^,^63^,^64^,^65^.

Exposure to male chemo-signals that trigger sexual behaviour significantly enhances newborn cell integration in female mice^10^,^11^,^13^, and importantly the ablation of OB neurogenesis through chemical^11^,^13^, genetic^35^ or physical irradiation^36^ approaches always leads to abnormal socio-sexual behaviours in male and female mice^17^. These data strongly suggest that the number of newborn neurons that are integrated in the OB region, and particularly in the AOB, is critical for the optimization of pheromone-elicited reproductive responses. Although sex behaviour involves a complex interaction and activity of multiple steroid-sensitive brain circuits, in this context our results add two new important pieces of evidence: i) an increase in the integration of newborn neurons in the AOB drives attraction/preference for male cues (influencing the activity of downstream VNS circuits) independently of the genetic sex of mice; and ii) this (pheromone-elicited) increase depends strictly on low circulating TST levels, a condition typical of females^66^.

The interaction of gonadal hormones and adult-generated neurons in promoting ‘appropriate’ opposite-sex attraction also sheds new light on the way sex differences in the anatomy and function of the VNS are achieved^60^,^67^, at least in the circuits involved in the regulation of pheromone-evoked reproductive responses. Based on our data, we propose that sexual dimorphism in adult VNS circuits is sustained by adult neurogenesis, through which a diverse number of newborn neurons, key elements for sensory perception, are integrated based on sex-specific internal (gonadal hormones) and external cues (pheromones). Although we cannot rule out the possibility that this mechanism could be a peculiar feature of mice because sex hormones differentially modulate adult neurogenesis depending on species, sex and age^68^,^69^, our study revealed a key role of adult neural plasticity in the gonad-dependent regulation of sensory cue-elicited sex behaviours.

## Methods

### Animals

The experiments employed adult, 3- to 5-month-old male and female mice. The Semaphorin 7A homozygous mutant constitutive knockout mice (C57BL6/J strain) were generated by Alex Kolodkin (John Hopkins University School of Medicine) and provided by Prof. Pasterkamp (Utrecht University). Mutant phenotypes were obtained by crossing heterozygous mice, and wt mice from the same litters were used as the control group^18^. After DNA was isolated from tail samples, genotypes were determined using PCR amplification. Animals of the same sex were maintained 4–6 per cage in rooms with a 12:12 light/dark cycle, with standard diet and water provided ad libitum. The male subjects that were used for behavioural analysis were kept in isolation for the duration of the behavioural experiments. All experimental procedures were conducted in accordance with the European Communities Council Directive of November 24, 1986 (86/609/EEC), Recommendation 18/06/2007, Dir. 2010/63/UE, and with the University of Turin’s institutional guidelines on animal welfare (DL116/92). The experiments were also approved by the Italian Ministry of Health and the Bioethical Committee of the University of Turin (Protocol Number DGSAF0007085-A05/04/2013). All experiments were designed to minimize the number of animals used.

### 5-Bromo-2′-deoxyuridine (BrdU) treatment

To identify newly generated cells in the OB, mice were intraperitoneally injected with BrdU in 0.1 M Tris (pH 7.4) twice a day (delay = 8 h, 100 mg/kg body weight) and killed 28 days later for to evaluate neuronal survival. Cell proliferation in the SVZ-RMS in the clean bedding condition was analysed 2 hours after a single intraperitoneal injection of BrdU (0.1 M Tris, pH 7.4; 100 mg/kg body weight).

### Tissue preparation

Mice were deeply anesthetized via an intraperitoneal injection of a 3:1 ketamine (Ketavet; Gellini, Italy):xylazine (Rompun; Bayer, Germany) solution. All of the animals were transcardially perfused with a 0.9% saline solution followed by cold 4% paraformaldehyde (PFA) in 0.1 M phosphate buffer (PB), pH 7.4. The brains were removed from the skull and post-fixed for 4–6 h in 4% PFA at 4 °C. Post-fixing was followed by a cryopreservation step with a 30% sucrose solution in 0.1 M PB pH 7.4 at 4 °C. The two hemispheres were separated and embedded in OCT (Bio-Optica) and then frozen and cryostat sectioned. Free-floating parasagittal and coronal sections (25 μm) were collected in multi-well dishes to provide representative series of the AOB and MOB, respectively. The sections were stored at −20 °C in an antifreeze solution (30% ethylene glycol, 30% glycerol, 10% PB: 189 mM NaH_2_PO_4_, 192.5 mM NaOH; pH 7.4) until use.

### Immunohistochemistry

After the sections were rinsed in PBS to remove the antifreeze solution, they were incubated for 24 h at 4 °C in primary antibodies diluted in 0.01 M PBS, pH 7.4, 0.5% Triton X-100, and 1% normal sera that matched the host species of the secondary antibodies. The following primary antibodies were used: anti-BrdU, rat IgG monoclonal, dilution 1:5000 (ABC), 1:1000 (IFL), AbD serotec, Bio-Rad Laboratories, code number OBT0030CX (Liu *et al*., 2009); and anti-cfos, rabbit IgG polyclonal, dilution 1:10000 (IFL), Santa Cruz biotechnologies, CA, USA, code number sc52^11^. For BrdU immunostaining, the sections were pre-treated with 2N HCl for 30 min at 37 °C for antigen retrieval and neutralized with borate buffer, pH 8.5, for 10 min. For the avidin-biotin peroxidase method, the sections were incubated for 1 h at room temperature in a biotinylated secondary antibody (anti-rat IgG, anti-goat IgG; Vector Laboratories, Burlingame, CA) diluted 1:250 in 0.01 M PBS, pH 7.4, followed by incubation with the avidin-biotin-peroxidase complex (Vector Laboratories). To reveal immunoreactivity, we used 0.015% 3,3′-diaminobenzidine and 0.0024% H_2_O_2_ in 0.05 M Tris-HCl, pH 7.6. After the sections were adhered onto gelatin-coated glass slides, they were dehydrated and mounted in Sintex (Nuova Chimica, Cinisello Balsamo, Italy).

For immunofluorescence double-staining, the sections were incubated in a mixture of primary antibodies and appropriate blocking sera for 24 h at 4 °C, then incubated with appropriate fluorochrome-conjugated secondary antibodies (Cy3-conjugated secondary Ab, 1:800; 488-conjugated secondary Ab, 1:400; Jackson ImmunoResearch Laboratories, USA) and/or biotinylated secondary antibodies (1:250; Vector Laboratories, Burlingame, CA, USA), and finally incubated with avidin-FITC (1:400, Vector Laboratories, Burlingame, CA, USA). The sections were then coverslipped with the anti-fade mounting medium Dabco (Sigma) and analysed with a laser scanning LAS AF Lite confocal system (Leica Microsystems).

### Familiarization with male bedding

The experiment was carried out by defining eight groups of animals characterized by a specific combination of genotype (Sema7A ko or wt), gender (male or female) and treatment (exposure to male-soiled bedding or clean bedding).

The groups exposed to male-soiled bedding (familiarized groups) were exposed to a mixture of soiled beddings, collected from the cages of C57BL6/J stud males, from day 7 to day 14 after BrdU injection. The soiled bedding was renewed daily. The control groups (unfamiliarized animals) were kept in a separate room from day 0 to day 28 after BrdU injection and treated with clean bedding in the same way. In all animals (the familiarized and unfamiliarized groups), the stimulus (soiled bedding) was presented focally (30 min) 90 min before the animals were perfused to detect c-fos activation.

### Olfactory preference test

For all behavioural tests, the animal subjects were coded so that the investigator was blind to the phenotype of each animal. During the social olfactory preference test assessment, the wt and Sema7A ko mice (males and females) were first exposed to urine from an adult C57BL6/J wt stud male and oestrus female, respectively, for 30 min. Then, after 30 min in clean bedding, the mice were presented with the same urine samples, which were delivered on a piece of filter paper placed within a petri dish (enabling direct contact with the source) or in a drilled box (enabling contact only with the volatile compounds). Male and female urine samples were placed on opposite sides of the cage, equidistant from the cage walls. Trials lasted 10 min, during which mouse behaviour directed towards the two urine sources was recorded. The amount of time spent sniffing the petri dish or the drilled box in which a test urine sample was presented was used as an indication of the mouse’s interest in gaining further information from a scent source. The tests were conducted in the animals’ home cages to minimize both manipulation and exposure to external stimuli. For each test, approximately 50 μl of either male or female urine was put on each piece of filter paper. The duration of the sniffing of the tested mouse was quantified using JWatcher Behavioral Software (UCLA, USA).

### Individual preference test

Sex preference was assessed using a three-chamber cage^31^. The dividing walls were made from clear Plexiglas, with an open middle section, allowing free access to each chamber. Two mice, one male and one female, were placed inside two identical wire cup-like containers with removable lids that were large enough to hold a single mouse. These containers were placed vertically inside the apparatus, one in each side chamber. Each container consisted of metal wires to allow for air exchange between the interior and exterior of the cylinder but was small enough to prevent direct physical interactions between the animal on the inside with the animal on the outside. The main principle of this test is based on a subject mouse’s free choice to spend time in contact with the mice in the containment cups. Before starting the test, a female mouse in oestrus was chosen to be held in one of the containers. To determine the ongoing oestrous cycle phase of the female mice used in the behavioural experiments, vaginal cytology was evaluated through optical microscope observations of smears. Samples were collected non-invasively via glass Pasteur pipettes after flushing with a saline solution. Adult C57BL6/J wt and Sema7A ko male mice that were previously cage-isolated for at least one week were tested. The morning before the test, the mice were accustomed to the empty cage during a 12-min acclimation period. The amount of time spent in direct contact with the cup-like container (containing a male or female mouse) was evaluated over a 15-min test period, and the mouse’s social odour preference was defined as the propensity to spend more time sniffing one containment cup than the other. The duration of direct (active) contact between the tested mouse and the containment cup was quantified using JWatcher Behavioral Software (UCLA, USA). The percentage of time spent investigating one of the two animals (male or female) relative to the total amount of time spent investigating both animals (sniffing period) was evaluated.

### Habituation/dishabituation test

Odour habituation/dishabituation was used to measure MOE-mediated olfactory discrimination^33^,^34^. Briefly, mice were familiarized with a first odour (habituation odour) in four successive sessions and then exposed once to a novel odour (dishabituation odour). Each session was 1 min long and was followed by a 10-min inter-session interval. Olfactory discrimination was analysed using two different odours, acetophenone (00790, Sigma) and octanal (O5608, Sigma), which were diluted 10^−3^% in mineral oil (M3516, Sigma) and delivered via patches of filter paper. Twenty microliters of odour was applied to each filter paper, which was then placed in a petri dish. The two odours were both alternatively tested as novel stimuli (during dishabituation) in separate sessions. Mouse sniffing activity was quantified using JWatcher Behavioral Software (UCLA, USA).

### Odour detection threshold test.

The test was performed as previously described^34^ with minor modifications. Five days prior to the test, mice (Sema7A ko and wt) were single housed in their home cages, then used as testing chambers. During the test, each animal was exposed to two drilled boxes, placed at the opposite sides of the cage, containing a piece of filter paper (1,5 × 1,5 cm) soaked one with 25 μl of odorless mineral oil (M3516, Sigma), as a control, and the other with 25 μl of octanal (O5608, Sigma), diluted in mineral oil. Sniffing time was measured for four concentrations of the odour (10^−7^, 10^−5^, 10^−4^, 10^−3^) tested in separate sessions in ascending order. Each session was 5 min long and was separated by 15 min intersession interval. Each session was video-recorded, and the time the animals spent sniffing the box, defined as a nasal contact with the dish within a 0.5 cm distance, was subsequently measured by BORIS software^70^. For statistical analysis, an ‘odour preference’ index was calculated, as the ratio between the time spent investigating the odour, and the total sniffing time (odour plus mineral oil). The values between 0.50 and 1.00 were indicative of a preference for the odour, compared to mineral oil.

### Castration procedure

Adult (3 months old) wt C57BL6/J mice were deeply anaesthetized with a solution of ketamine and xylazine, and using aseptic procedures, both testes were removed by a small incision in the scrotum. The spermatic cord was then blocked with a silk suture to prevent haemorrhage during dissection of the testis. The mice were then allowed to recover for 3 weeks before further treatments.

### Testosterone treatment

For the analysis of adult neurogenesis, the TST treatment was applied in one group of intact Sema7A ko males and in castrated wt C57BL6/J males (24 h after BrdU administration, see Figs 5A and 6A). Another group of Sema7A ko males was treated with TST for the analysis of Ar expression. The treatment consisted of daily subcutaneous injections of testosterone propionate (1 mg/day), diluted to 10 mg/ml in sesame oil^42^ for 16 consecutive days. Control animals received sesame oil treatment only. During the TST/oil treatment, the animals underwent behavioural analyses and familiarization with stud-male bedding. Ten days after the behavioural analyses, two additional daily pulses of TST/oil were given before focal bedding presentation and sacrifice.

### Testosterone assays

Trunk blood was collected directly from the mouse’s heart into a sterile eppendorf tube with a needle and left on ice until centrifugation; the plasma was frozen and stored at −80 °C until use. Plasma TST was measured using a sensitive testosterone rat/mouse ELISA (Demeditec Diagnostics REF DEV9911) based on the principle of competitive binding. The lowest analytically detectable level of TST that can be distinguished from the Zero Calibrator is 0.066 ng/ml. The intra- and interassay coefficient of variation was 8.06% and 9.3%, respectively.

### Quantitative RT-PCR analyses

For gene expression analyses, OBs and POA were extracted and fresh-collected from wt and Sema7A ko male mice (treated with TST and untreated). Total mRNA was extracted from these bulbs using the RNeasy^®^ Lipid Tissue Mini Kit–74804 (QIAGEN) and reverse transcribed using SuperScript^®^ III Reverse Transcriptase (Life Technologies). A linear preamplification step was performed using the TaqMan^®^ PreAmp Master Mix Kit protocol (Applied Biosystems). Real-time PCR was carried out on a StepOnePlus TM Real Time-PCR System (Applied Biosystems) using the following exon-boundary-specific TaqMan^®^ Gene Expression Assays (Applied Biosystems): Er1 (Mm00433149_m1), Er2 (Mm00599821_m1), and Ar (Mm00442688_m1). Relative gene expression levels were normalized against the mRNA levels of the housekeeping genes β-actin (Mm01205647_g1) and RN18S (Mm03928990_g1). Quantitative real-time PCR was performed using TaqMan Low-Density Arrays (Applied Biosystems) on an Applied Biosystems 7900HT thermocycler using the manufacturer’s recommended cycling conditions. Gene expression data were analysed using ExpressionSuite Software v1.01.

### Cell count and statistical analysis

Cell counts and image analyses were performed on a Nikon microscope equipped with a computer-assisted image analysis system (Neurolucida software; MicroBrightField, Colchester, VT) and on a Leica DM600 CS LAS AF Lite confocal microscope (Leica Microsystems). Confocal image z-stacks were captured through the thickness of the slice at 1-μm optical steps. These images were used for cell counting or assembled into extended-focus photographs. Brightness, colour, and contrast were balanced and assembled into panels with Inkscape (Free vector graphics editors). All cell counts were performed blind to the genotype and/or the treatment.

In the AOB, the number of all newborn neurons was established by counting peroxidase/DAB-stained or fluorescence-labelled BrdU-positive nuclei in 25-μm-thick parasagittal sections. Two series (n = 7/8 sections/animal) representing the whole AOB were used for each animal. Cells were counted in the AOB granule cell layer. The area of each examined section was measured with Neurolucida software. Cell densities were expressed relative to the volume of the entire AOB granule cell layer; this volume was obtained by multiplying the area measurements by the mean section thickness (25 μm).

The percentage of double-labelled c-fos/BrdU-positive cells in the AOB granule cell layer was established by counting labelled cells in parasagittal sections (25 μm thick) with a 40x objective. One series (n = 3–4 sections/animal) representing the whole AOB was used for each animal. In each section, all of the BrdU-positive cells were analysed for co-expression with c-fos, and ratios of double-labelled cells were determined.

In the MOB, the number of BrdU-positive nuclei was established by counting peroxidase/DAB-stained nuclei in three representative OB coronal (anterior, medial and posterior) or parasagittal (from lateral to medial) sections per animal using a systematic random sampling method. Newborn cells were quantified in the granule cell layer. Sampling was conducted by overlaying each section with a virtual counting grid (squares size of 80 × 80 μm). Cells were counted through the 25-μm thickness of the slice in one square of the grid (one of every two) through sequential translation of the counting frame until the area of interest was entirely covered (40x objective). This procedure allowed us to analyse about one-fourth of the area of interest. The number and position of each cell in the counted area were marked by the software. Cells contacting a line on the upper or left edge of the counting square were excluded from the counts, whereas those contacting the lower or right edge of the square were considered in the counts^71^. Cell density (number of labelled profiles/mm^3^) was calculated by multiplying the area measurements by the mean section thickness (25 μm) [Σ of sampled areas μm^2^ ×25 μm]. To evaluate cell proliferation in the SVZ-RMS region, newborn cells were quantified 2 hours after BrdU injection. Cell counting was performed on one-in-six parasagittal sections 25 μm thick. Eight different levels, along the rostro-caudal axis of the brain, were selected, based on stereotaxic atlas, and the total number of labelled cells was evaluated for each level. The number of BrdU-labelled cells was then multiplied by 6 to provide an estimate for the total number of newborn cells in the RMS-SVZ region.

For statistical analysis, unpaired student’s t-tests were used for simple 1:1 comparisons of parametric data, while the Wilcoxon-Mann-Whitney test was used for non-parametric analyses. One-way Anova were used for multiple comparisons in case of parametric data; Kruskall-Wallis Test was used in case of non-parametric data (Supplementary Figures).

## Additional Information

**How to cite this article**: Schellino, R. *et al*. Opposite-sex attraction in male mice requires testosterone-dependent regulation of adult olfactory bulb neurogenesis. *Sci. Rep.* **6**, 36063; doi: 10.1038/srep36063 (2016).

**Publisher’s note:** Springer Nature remains neutral with regard to jurisdictional claims in published maps and institutional affiliations.

## Supplementary Material

Supplementary Information
