# Supplementary material for: Opposite-sex attraction in male mice requires testosterone-dependent regulation of adult olfactory bulb neurogenesis
Source: Sci Rep. 2016 Oct 26;6:36063. doi: 10.1038/srep36063 (PMC5080553; doi:10.1038/srep36063)
Supplement: Supplementary Information [file srep36063-s1.pdf]

Supplementary Figures

Opposite-sex attraction in male mice requires testosterone-dependent regulation of adult olfactory bulb neurogenesis

Roberta Schellino[1,2], Sara Trova[1,2], Irene Cimino[5], Alice Farinetti[4], Bart C. Jongbloets, R. Jeroen Pasterkamp[7], Giancarlo Panzica[2,3,4], Paolo Giacobini[5,6], Silvia De Marchis§[1,2,3] and Paolo Peretto*§[1,2,3]

[1]Department of Life Sciences and Systems Biology, University of Turin, Turin I-10123, Italy

[2]NICO – Neuroscience Institute Cavalieri Ottolenghi, University of Turin, Turin I-10125, Italy

[3]Neuroscience Institute of Turin (NIT), University of Turin, Turin I-10125, Italy

[4]Department of Neuroscience, University of Turin, Turin I-10125, Italy

[5]Inserm, UMR-S 1172, Laboratory of Development and Plasticity of the Neuroendocrine Brain, F-59000 Lille, France

[6]Univ. Lille, UMR-S 1172 - JPArc - Centre de Recherche Jean-Pierre AUBERT Neurosciences et Cancer, F-59000 Lille, France

[7]Department of Translational Neuroscience, Brain Center Rudolf Magnus, University Medical Center Utrecht, 3584 CG11 Utrecht, The Netherlands.

§These authors jointly supervised this work.

Running title: AOB neurogenesis and opposite-sex attraction

Keywords: adult neurogenesis, AOB, testosterone, sexual dimorphism, sexual attraction, reproduction

*Corresponding author:

Paolo Peretto, PhD,

Associate Professor, University of Turin

Dept. Life Sciences and Systems Biology (DBIOS),

Via Accademia Albertina, 13 - 10123 Torino -IT

Neuroscience Institute Cavalieri Ottolenghi (NICO)

Regione Gonzole, 10 - 10043 Orbassano (To) - IT

tel. +39-011670-4673/ -6605

email: paolo.peretto@unito.it

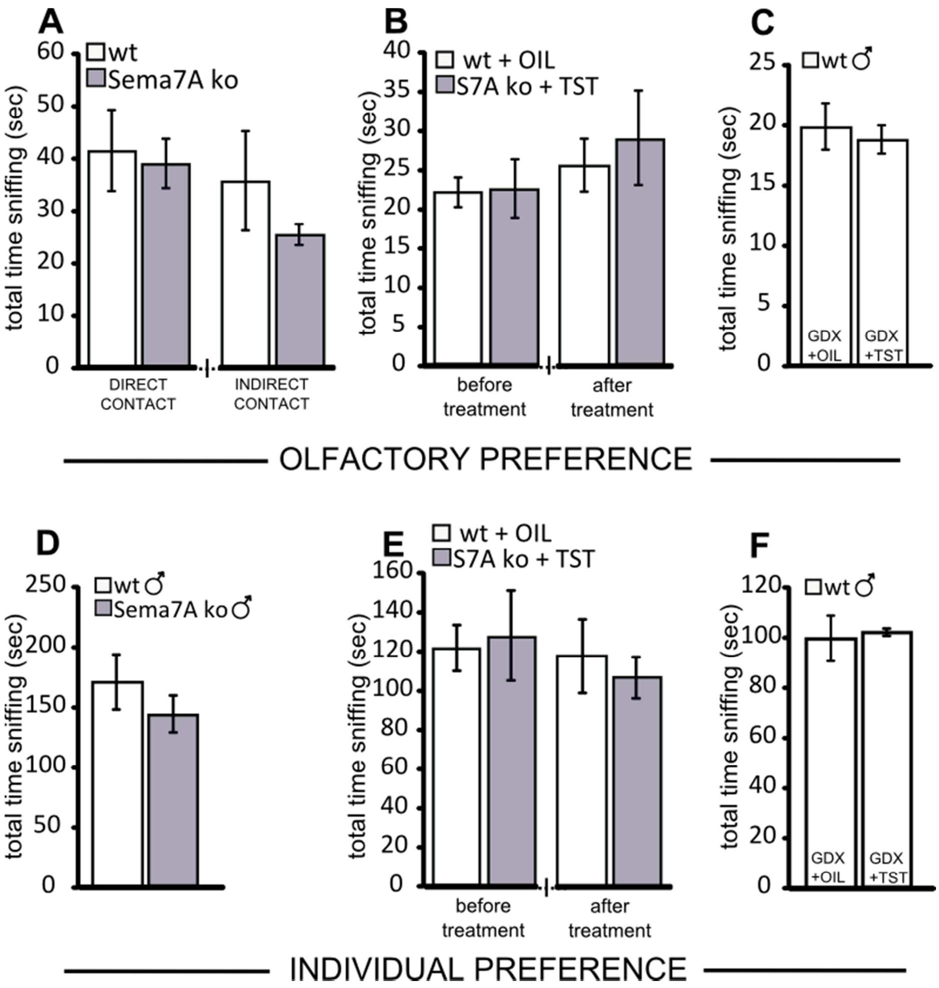

Supp. Figure 1: Total time of sniffing in olfactory and individual preference tests in wt and Sema7A ko males. The total amount of time (sniffing period) was evaluated by considering the whole time spent by each animal sniffing/interacting with scents /individuals of both sexes (male plus female odors/individuals). No significant differences (P>0.05) are evident in the olfactory preference test between Sema7A ko and wt males in all the considered conditions: (A) direct and indirect contact with the pheromonal source in intact males (wt and Sema7A ko animals); (B) before and after treatment with oil (wt) and testosterone (TST, Sema7A ko); (C) in oil- and TST-treated wt gonadectomized (GDX) males (n= 8 each group; Unpaired Student's t-test, P>0.05). (D-F) No significant differences (P>0.05) between the above-indicated groups are also visible when the total time of sniffing was referred to the social interaction assay (n= 8 each group; Unpaired Student's t-test, P>0.05). The values shown are the mean±s.e.m.

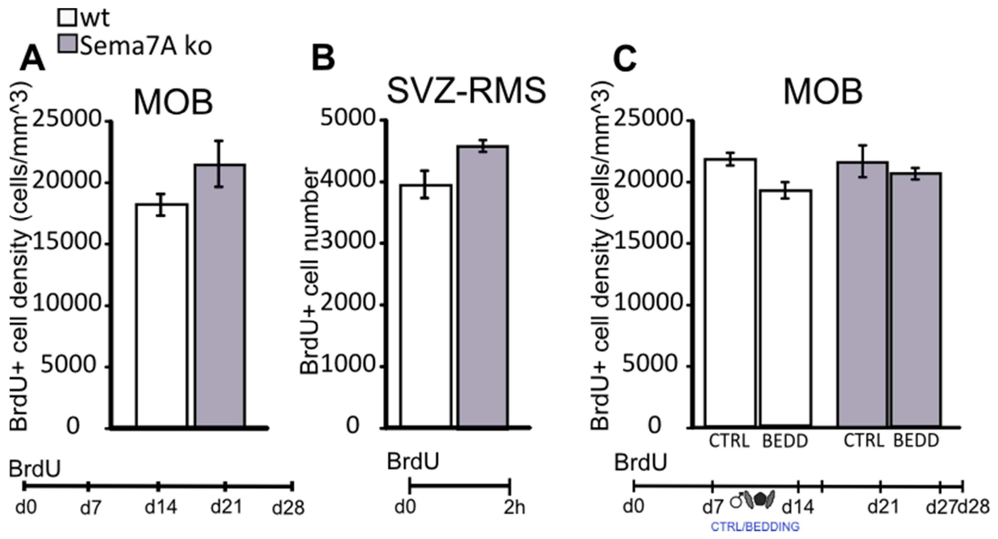

Suppl. Figure 2: Adult neurogenesis in the MOB and SVZ-RMS of Sema7A ko and wt males. (A, B) Clean bedding condition, (C) Male-bedding exposure. A) Newborn cell survival evaluated 4 weeks after BrdU injection in the MOB GrL shows no significant difference between wt and Sema7A ko males in the density of BrdU+ cells (n=4 each group; Unpaired Student's t-test; P>0.05). (B) No significant difference in the density of BrdU+ neurons is evident between Sema7A ko (n=4) and wt (n=5) males two hours after a single BrdU pulse (unpaired Student's t-test, P>0.05). (C) Cell survival in the MOB after 1 week-exposure/familiarization to male bedding/pheromones. After 28 days from BrdU injection, no significant differences are evident in newborn neurons cell density between control and male-bedding familiarized groups, in both wt and Sema7A ko males (n=4 each group; One-way Anova; P>0.05). Abbreviations: MOB: main olfactory bulb; SVZ-RMS: subventricular zone-rostral migratory stream; CTRL: control group; BEDD: bedding group; S7A: Sema7A; BrdU: bromodeoxyuridine. The values shown are the mean±s.e.m.

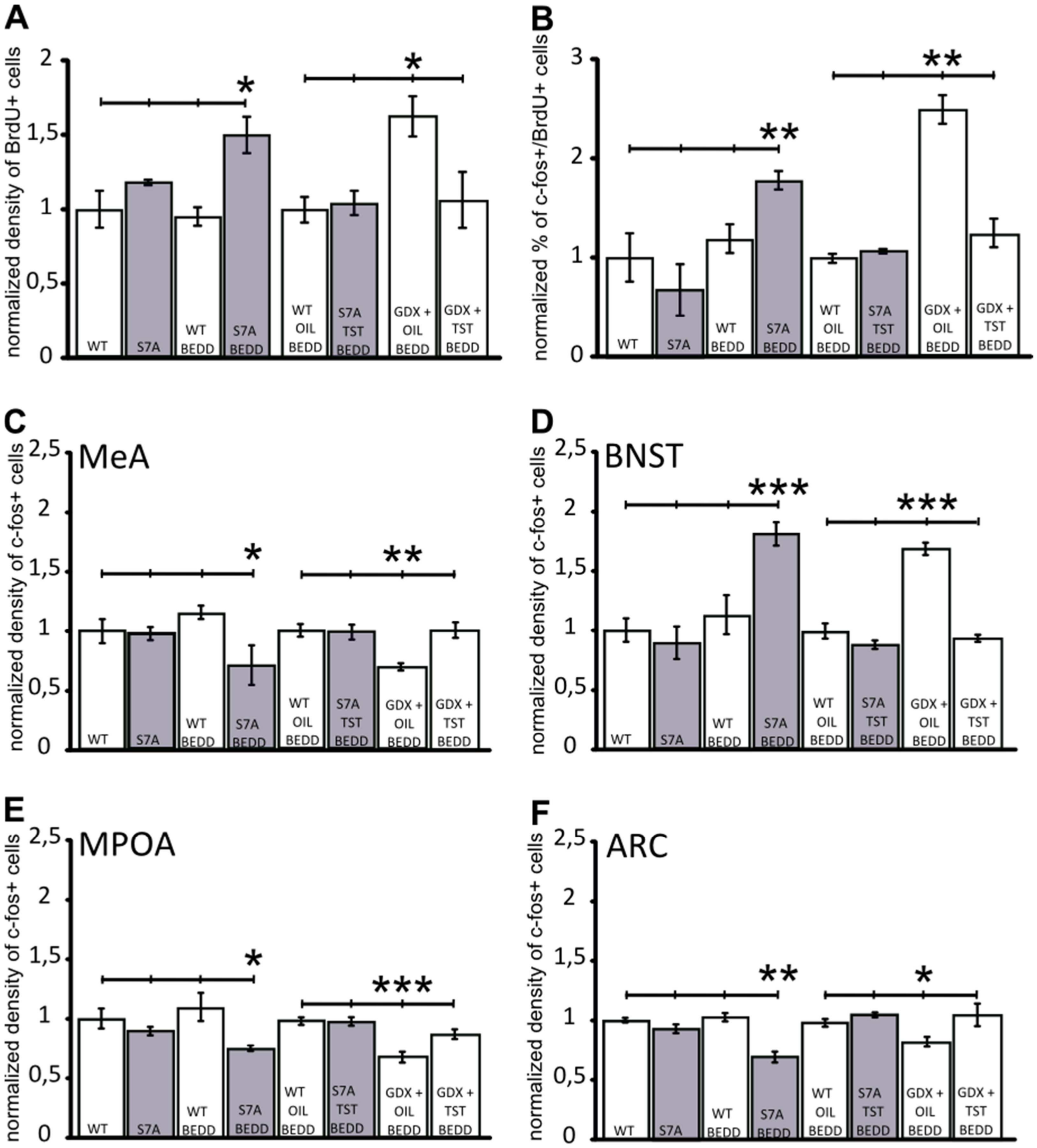

Suppl.Figure 3: Direct comparison of BrdU cell density and c-fos expression along the vomeronasal pathway in Sema7 ko and wt oil-and TST-treated male mice, in the clean bedding condition and after male bedding exposure. The data, coming from different experiments, have been normalized to wt intact/oil tretated animals (=1). Both the BrdU cell density in the accessory olfactory bulb (AOB) (A) and the c-fos expression in AOB newborn neurons (B) and in cells of other vomeronasal nuclei (C-F) clearly show similar patterns for the Sema7A ko intact males and wt gonadectomised (GDX) oil-treated males, after male bedding exposure. Similarly, same patterns are also visible between Sema7A TST-treated mice and wt oil-treated or wt GDX-TST-treated males. (A) One-way Anova, *P<0.05; (B) Kruskall-Wallis Test, **P<0.01; (C-F)

One-way Anova,*P<0.05, **P<0.01, ***P<0.001. Abbreviations: GDX: gonadectomy; TST: testosterone; BEDD: bedding; MeA: medial amygdala; BNST: bed nucleus of stria terminalis; MPOA: medial preoptic area; Arc: arcuate nucleus; BrdU: bromodeoxyuridine. The values shown are normalized mean±s.e.m.
